# The Progressive Public Measures of Saudi Arabia to Tackle Covid-19 and Limit Its Spread

**DOI:** 10.3390/ijerph18020783

**Published:** 2021-01-18

**Authors:** Anwar A. Sayed

**Affiliations:** 1 Department of Medical Microbiology and Immunology, Faculty of Medicine, Taibah University, Medina 42353, Saudi Arabia; dsayed@taibahu.edu.sa; 2 Department of Surgery and Cancer, Imperial College London, London SW7 2AZ, UK

**Keywords:** Covid-19, curfews, health protection, Saudi Arabia, social distancing

## Abstract

Since the beginning of the global novel coronavirus disease (Covid-19) pandemic, the response of governments has varied significantly across the world. This was one of the main reasons behind the difference in the disease fatality rates between countries. In this study, the public progressive measures taken in Saudi Arabia (SA) are described in detail. This is a descriptive study in which measures were retrieved via the government official news agency—Saudi Press Agency (SPA). The total number of Covid-19 cases and its fatality rate were obtained/calculated from the Saudi Ministry of Health (MoH) official Covid-19 port, as well as the WHO COVID-19 dashboard. SA took active measures in order to interrupt the spread of Covid-19 which were strict, rapid, and progressive in nature. Social distancing was at the heart of almost every measure taken by the government. The main themes of these interventions are as follows: travel bans, suspending religious activities, closure of non-essential shops, enforcing changes at workplaces, and imposing curfews. This was followed by the gradual return to working life with various precautions to prevent a spike in the number of cases. The imposed measures in SA succeeded in reducing the burden of Covid-19 and its fatality rate. Comparatively, the fatality rate in SA was significantly lower compared to developed countries with better healthcare infrastructure such as the United States and United Kingdom.

## 1. Introduction

Since the appearance of the novel corona virus disease (Covid-19) cases in Wuhan and its spread across China [1,2], governments around the world closely observed the situation. Covid-19 continued to spread rapidly across countries making it a ‘Global Pandemic’ as declared by the World Health Organization (WHO) [3]. However, no immediate actions or preventative measures were taken by most countries and their response to the pandemic varied significantly.

Studies have attempted to assess the usefulness of such preventative measures to limit the spread of the Covid-19 infection. Modeling studies have demonstrated that the most effective measure of reducing both the transmission and new cases, as well as reducing case fatalities, was quarantine in combination with other measures such as wearing face masks and travel restrictions [4,5].

Saudi Arabia (SA) was one of the earliest countries to respond to the pandemic by applying a series of public progressive measures. This article will present the public measures taken in SA and describe how these measures were responsive to the change in the disease infection rates. Additionally, it will highlight how SA’s figures preceded some developed countries like United States (US) and United Kingdom (UK).

## 2. Materials and Methods

This is a descriptive study in which all interventions in SA were retrieved from the official state news agency, Saudi Press Agency. In addition, the Ministries of Health and Interior announced their measures through their official channels i.e., websites and social media platforms. Measures that were rumored or presented in unofficial sources were not included in this study.

Information search was conducted in PubMed, Ovid MEDLINE, and Embase databases. The search terms used were ‘Saudi Arabia’ AND ‘Covid-19′ OR ‘Sars-Cov-2′ AND ‘Public measures’. Studies published between February and October 2020 were included in this study. The total number of confirmed Covid-19 cases, as well as number of deaths due to Covid-19, for the period between February and December 2020 were obtained from the officially announced data on the WHO COVID-19 dashboard, as well as the Worldometer Covid-19 Portal [6,7]. There was no discrepancy between the Covid-19 data of the Saudi population presented on the Saudi Ministry of Health portal and the WHO COVID-19 dashboard. Therefore, the WHO COVID-19 dashboard was used to compare between SA and other countries.

## 3. Results

**Figure 1:**
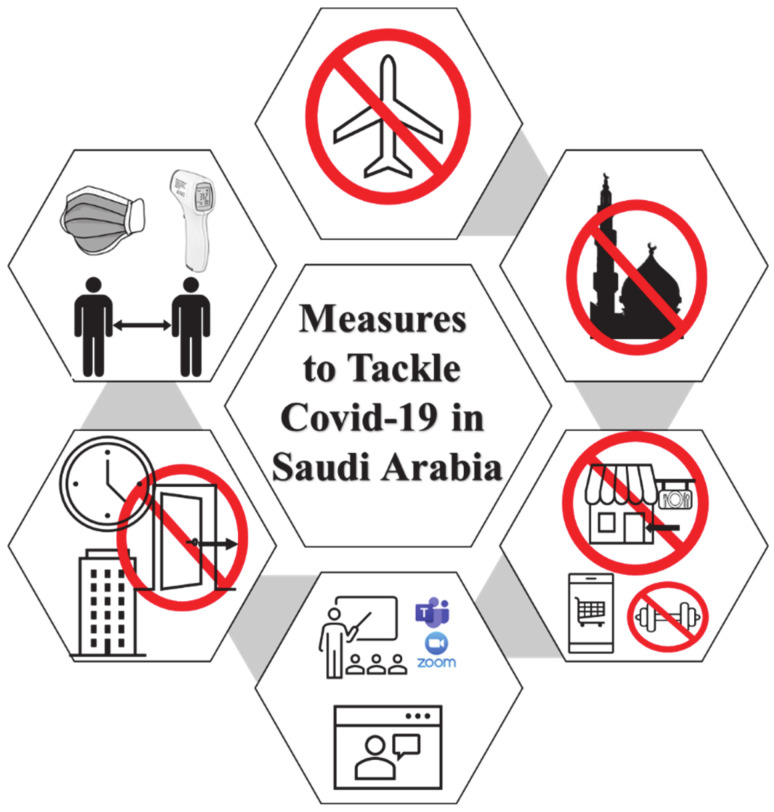
The measures taken to tackle Covid-19 in Saudi Arabia. The diagram summarizes the measures taken by the Saudi government to limit the spread of Covid-19. These measures include: travel bans, suspending religious activities, closure of non-essential shops, changes to workplace (including the switch to distance learning), and imposing curfews. Eventually, new regulations have been in place for a gradual safe return to the lifestyle prior to Covid-19. These guidelines include the mandatory facemask in public, measuring people’s temperature upon entering closed spaces, and other social-distancing measures.

Social distancing was at the heart of almost every measure taken by the Saudi government which aimed to reduce human-to-human transmission as much as possible. The main themes of these interventions are as follows: travel bans, suspending religious activities, closure of non-essential shops, enforcing changes at workplaces, and imposing curfews (Figure 1). Eventually, new regulations have been in place for a gradual safe return to the lifestyle prior to Covid-19. These guidelines include the mandatory facemask in public, measuring people’s temperature upon entering closed spaces, and other social-distancing measures.

### 3.1. Travel Bans

The earliest measure that SA took was suspending several international flights of all incoming/outgoing travels to/from several countries. SA issued its first travel ban at the end of January to all flights to and from China. All Saudi students in China and Korea were evacuated back to SA and were quarantined for 14 days before being released to their families. As the number of positive cases continued to soar globally, SA suspended issuing visas to all visitors, as well as incoming international flights by the end of February. Despite all these measures, SA recorded its first case of Covid-19 on the 2nd of March to a citizen who just returned from Iran through Bahrain. As a result, SA further expanded the travel bans to include all international travels by all modes of transportations.

### 3.2. Suspending Religious Activities

Saudi Arabia hosts 2 of the holiest sites in the Islamic world: the Grand mosques and the Prophet mosque in Makkah and Madinah, respectively. The Grand mosque and the Prophet mosque accommodate over 1 and 2 million visitors respectively, during peak times [8,9]. These 2 sites are mostly visited between the beginning of the 8th and the end of the 12th month of the Islamic calendar, which coincided with the months of March and July of 2020. The country receives over 10 million visitors annually for the purpose of Umrah and pilgrimage [10,11]. On the 26th of February, the government stopped issuing Umrah visas to all foreign visitors, and was followed by suspending the Umrah for everyone, regardless of their residence.

Besides these 2 holy mosques, SA hosts over 84,500 mosques all over the country in which people aggregate to pray 5 times a day [12]. In these prayers, people line-up in very close proximity to each other. As a result, such environments would be a very fertile medium for the mass viral transmission between people. These mosques are particularly busy during the Friday prayer, achieving almost full capacity in most cases and even praying outside the holy mosques. Between these prayers, small-group classes about Islam and Quran take place in these mosques. The government suspended all praying and teaching activities in all mosques, in order to prevent all forms of gatherings.

### 3.3. Closure of Non-Essential Shops and Activities

On the second week of March, the government ordered the closure of all shopping centers across the country. All coffee shops and restaurants were also ordered to continue its services via takeaways, drive-thru, or home-delivery and thus preventing any customers from entering the premises. Home delivery workers were instructed to wear face masks and disposable gloves throughout the delivery process and to leave the goods to be picked by customers from a distance. Gyms and sport clubs were also closed temporarily as they are considered a major source of infection transmission. These measures were associated with the temporary closure of all event halls e.g., wedding halls, as well as sports and entertainment events.

Essential shops such as supermarkets, pharmacies, and gas stations were excluded from any restrictions and continued business as usual, and home delivery of goods during curfews.

### 3.4. Enforcing Changes at Workplaces

As the number of Covid-19 cases continued to rise, new workplace regulations were legislated and put into effect immediately. All in-class educational activities in nurseries, schools, and universities were suspended and changed into distance learning using online platforms.

Other sectors were instructed at first to suspend the fingerprint attendance system and to reduce the staff load at workplaces to a maximum of 25% of the workforce, if needed. Many organizations stopped receiving customers in their offices and switched to online means to deliver their services.

### 3.5. Imposing Curfews

On the 22nd of March, the Saudi government imposed a nation-wide 11-h curfew from 7 p.m. to 6 a.m. These curfews entail banning people from being on the street in any form i.e., walking, cycling, or driving. Travelling between cities was also suspended.

The authority of each administrative region (province) was permitted to take further measures suitable to its own circumstances. Al-Qatif, a city in the Eastern region of SA, was one of the first cities to enforce a complete lockdown in which people could not go in or out of the city. This was due to the exponential increase in Covid-19 cases due to its locals visiting Covid-19-infested countries such as Iran. Makkah and Madinah were the first cities in the country in which the curfew duration was extended for an additional 4 h.

As the number of cases continued to increase, all neighborhoods across the nation were locked down throughout the day and thus preventing people from moving between neighborhoods of the same city. This was in addition to the curfew hours.

The application of these curfews was achieved by police divisions, the national guard, and army using roadblocks, automated cameras, and patrols. The violation of these curfews was heavily punished with a penalty of SAR 10,000 (~USD 2667) for first timers which doubled upon repeated violation. Additionally, a 20-day imprisonment, and deportations for foreign residents, who were repeated curfew violators.

Location-based mobile applications were introduced for granting temporary permissions in cases of emergency.

Some keys workers were exempted from these curfews during their working hours. Those include law-enforcing agents, medical personnel, delivery drivers, as well as pharmacies and gas station workers.

### 3.6. “We All Return Cautiously”

Towards the end of May, the government with its different agencies launched a major campaign titled “We All Return Cautiously”. This campaign was accompanied by a set of established guidelines on the gradual return to what was considered as normal life i.e., prior to the Covid-19 pandemic. General guidelines included mandatory wearing of face masks in public, measuring people’s temperature prior to their entry to public places, setting a maximum capacity for closed locations, ensuring social-distancing, and minimizing the handling of items by multiple people where possible.

Wearing face masks has become mandatory when going in public, and violators are penalized with a SAR 1000 (~USD 267) fine. This has also become an entry requirement to any closed place.

Public and workplaces were instructed to have a designated person who measures people’s temperatures using a contactless thermometer prior to their entry. Bigger shopping centers used thermal cameras for such purpose. Anyone who was identified to have fever was isolated and local health authorities were called to take the necessary measures to examine, isolate, and treat him/her if needed.

Another important measure was limiting the number of people in a closed area based on its surface area. A surface area of 1.5 m^2^ was determined for each person so that shops could determine their maximum capacity of people at any given time, including their staff.

At shops and supermarkets, the provision of hand sanitizers and disposable gloves for customers have become mandatory. Shops were also strongly advised to clean shopping baskets and trolleys after each customers’ use. The provision of a contactless payment option became obligatory across the nation. Additionally, floor markings were placed at shops entrances and at checkouts to ensure customers stand at safe distances from each other. At restaurants and coffeeshops, all reusable/washable items such as menus and utensils were replaced by digital menus and plastic or other disposable items.

## 4. Discussion

The Covid-19 pandemic has posed a huge challenge to countries around the world. The measures taken by countries to tackle it differed significantly according to their financial capacity and the confidence of the decision makers in science. Furthermore, the extent to which these measures were applied and enforced also varied between countries.

Since the beginning of the Covid-19 epidemic in China, health authorities in SA were closely monitoring the situation and putting together a contingency plan if it reached SA. When the first case of Covid-19 appeared in SA, the government was ready to enforce all the necessary public measures to protect the Saudi population from Covid-19 and slow down its spread, despite the lack of evidence-based information regarding its spread. These measures have two main aspects to them; they protect everyone who is currently in SA from any possible infections coming from abroad, and they instill certain protective behaviors gradually into the public conscious so they could follow it going forward.

The first measure taken by SA was banning direct travels between SA and China, the reported origin of the infection. However, this was not sufficient as travelers could still come into SA via alternative routes such as the Arabian Gulf states—by air or land. In fact, the first case of Covid-19 to enter SA was of a citizen returning from Iran through Bahrain. Many of the Shia Muslims in the Eastern region of SA visit Iraq and Iran for religious purposes. As Iran was one of the early countries to be badly affected by the virus [13], such transmission from Iran into SA was almost inevitable [14]. The ban on international travel, whether into or out of the country, was an important step so the authority could prevent the influx of cases, especially the asymptomatic ones from abroad. It has been argued that this measure is violating the International Health Regulation [15]. However, it was a complete international travel ban (to and from SA) and it was based on scientific evidence, suggesting it as an effective step in reducing the reproduction number i.e., viral transmission [16]. Additionally, a similar measure was taken by other countries such as China, UK, and US [17,18].

The transmission of the novel corona virus was reported to be droplet-based transmission [19]. Other case studies suggested alternative modes of transmission e.g., airborne, however, such proposal is still to be validated [20]. Therefore, the authorities considered both possibilities seriously and issued several measures accordingly.

Social distancing has been identified as the most important step in tackling Covid-19 [21]. Several simulation and modeling studies have demonstrated the importance of social distancing in limiting the spread of Covid-19 [22]. Religious tourism in SA is one of the main reasons millions of visitors come to SA annually. Umrah and Hajj visitors exceed a minimum of 100,000 visitors/pilgrims for any given instance. Additionally, SA hosts a rather huge number of mosques in which people gather to pray 5 times daily. The 2 holy mosques in Makkah and Madinah host a minimum of over 50,000 visitors for each prayer. The presence of an infected person in one of these sites would infect a huge number of visitors, starting a chain of infections similar to that of case no. 31 in Korea [23]. Therefore, closing all mosques and suspending the Umrah for everyone have become a necessity to contain the situation [24]. Religious tourism is one of the most import sectors of the Saudi national economy with a revenue, from the 2016 Hajj season only, of 21 billion US dollars [25], with an expected increase in the following years. Hence, by taking such drastic measures, the Saudi government has put the public health and welfare ahead of its financial gains.

People’s gatherings e.g., at educational establishments or shopping centers pose a great risk for viral transmission. SA suspended all face-to-face teaching and learning activities in the early stages of the pandemic in SA and transitioned to distance learning nationwide in accordance with the WHO recommendations [26]. Additionally, sports and entertainment activities were suspended. Coffee shops and restaurants were instructed to not allow dining in, and customers can only order from restaurants’ doors, drive-thru, or via home deliveries. As a result, many businesses have decided to close temporarily as it was not profitable to remain open as the number of customers decreased significantly. Many supermarkets and restaurants opted to home-delivery options as it was the only viable option for business in such difficult times. Although some businesses were negatively affected by the pandemic, home delivery services have provided ample job opportunities to citizens who may have been made redundant. Protective measures were also in place to ensure the safety of the home delivery workers, as well as their customers. The home delivery option was ideal for lowering the number of people at shops; however, it can also serve as a source of infection. If the precautions are not followed properly by the delivery personnel, this might put him at risk of acquiring the infection from customers, as well as spreading it to customers at their homes.

By far, curfews were the most effective measure in curbing the number of cases in SA. This can be observed as the number of daily new cases during curfews were in hundreds, and currently after lifting them off, cases are exceeding 2000 cases daily. SA was one of the first countries to take such a drastic measure to contain the infection. Other countries such as US followed SA’s footsteps by imposing curfews in highly infected cities [27]. Although the value and ethicality of these curfews could be argued, it seems to be the most effective method in enforcing social distancing and limiting public crowding. Importantly, the wellbeing of the citizens was not neglected at such times. Electronic permissions have been developed and were granted when needed e.g., medical emergencies, using a mobile application.

**Figure 2:**
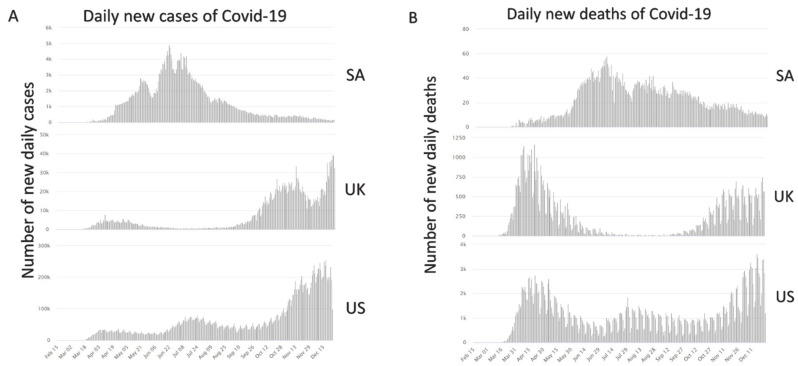
Daily new cases and deaths of Covid-19 in Saudi Arabia in comparison to UK and US. (**A**) The 3 graphs demonstrate the number of daily new cases in SA compared to UK and US. The peak number of new daily cases in SA is significantly less than UK and US. Such difference can be appreciated by the vast difference in the labeling of the Y axis. Similarly, (**B**) the peak number of daily deaths of Covid-19 infections in SA was significantly less than the UK’s and the US’s. Figures were retrieved from the Worldometers Covid-19 portal [7]. SA: Saudi Arabia; UK: United Kingdom; US: United States.

The strict application of these public precautions and protective measures have been successful in slowing down the spread of the disease and not overburdening the local healthcare system. These measures have positively influenced the public awareness and improved their day-to-day interactions, in light of Covid-19. The peak number of daily new cases of Covid-19 in SA was significantly lower than in UK and US (Figure 2A). Although the UK population is almost twice the Saudi population, the peak number of UK cases was almost 10 times the peak SA cases. Population wise, the US population is 10 times the SA population, however, the US peak number of daily cases was over 50 times the cases in SA. Furthermore, these measures prevented the occurrence of the second wave in SA, as compared to both UK and US. Similarly, the daily death rates in SA reached a peak of less than 60, whereas the number in UK and US were in hundreds and thousands, respectively (Figure 2B).

Additionally, the case fatality rate of Covid-19 was significantly lower in SA at the early phases of the diseases compared to other countries in the region such as Turkey and Iran, at 1.35% vs. 2.19% and 6.25%, respectively. Although these measures could not completely eradicate the virus from the country, it certainly helped in reducing the fatality rate from its early stages, despite the lack of effective treatments or vaccines. Additionally, the fatality rate of Covid-19 in SA went down from 1.35% in March to 1.01% in July [6]. The current fatality rate in SA (1.01%) is significantly better than some developed countries with better healthcare infrastructure such as US (3.53%), Canada (7.87%), UK (15.33%), and other European countries e.g., France (16.82%) [28]. A common theme between all these other countries was the lack of, delay, or lenient enforcement of measures to tackle Covid-19. Many of these countries issued guidance promoting social distancing and wearing face coverings. However, these were mostly made on a voluntary basis with no serious consequences for those who do not follow them.

No strict public measures were enforced in Sweden. The rationale behind such approach was the concept of herd immunity i.e., population-wide protection from an infection post vaccination or active exposure to an infection [29]. Another common justification was the lack of a model or data that allows analyzing the behavior of the COVID-19 infection rate was progressively reduced in relation to the measures taken [30]. This approach was considered successful initially as the number of new Covid-19 cases and its fatality rate were very low comparable with other European countries that enforced different precautions. However, Sweden was challenged with sudden increase in its cases and the fatality rate that burdened the country’s healthcare system [31].

In England, face covering has been made mandatory in indoor spaces like public transport, indoor transport hubs, and national health service (NHS) settings [32]. However, this measure was not enforced in public open-space areas such as parks and beaches. Furthermore, there were no effective restrictions on mass crowding in public. Therefore, the lack of face coverings along with mass crowding can be considered as a “recipe for disaster” in the context of Covid-19.

The country started a nationwide campaign called ‘We All Return Cautiously’ which reflects the nature of returning to the pre-Covid-19 lifestyle. Much has been learnt about the virus and the best measures to tackle it since its appearance. In keeping with the current evidence, SA has enforced some public measures such as face-coverings, maintaining social distancing, measuring people’s temperature before entering any closed buildings. Other unnecessary measures such as wearing disposable gloves and the use of personal protective equipment by the public were dismissed [33].

It is important to acknowledge that the low Covid-19 case fatality rate is not solely due to these public measures. SA, like other countries, has dedicated medical resources and interventions for tackling the pandemic which also contributed to the success of the Saudi experience with Covid-19. However, as discussed earlier, the medical infrastructure in SA is far preceded by other countries, and its detailed description is beyond the remit of this article.

## 5. Conclusions

SA implemented a series of progressive public measures to tackle Covid-19 from its early stages in the country. These measures were evidence-based, appropriate, in line with international recommendations, and were strictly enforced. The successful application of these measures in SA led to improving the public awareness and behavior. This in turn led to SA having lower number of new cases than predicted in its early phases. Additionally, it led to one of the lowest Covid-19 case fatality rates, compared to other developed countries with a better healthcare infrastructure. Until the development and provision of an effective Covid-19 vaccine and nationwide immunity against it, countries which are badly affected by the virus should follow the Saudi experience in dealing with Covid-19.

## Data Availability

Publicly available datasets were analyzed in this study. This data can be found here: https://www.who.int/emergencies/diseases/novel-coronavirus-2019/interactive-timeline?gclid=CjwKCAiAl4WABhAJEiwATUnEF5RRrhA4IGUZEpKYWv7jCsg3jdx1A_i1IROWObN_C3YIL7JkYNP5yRoC8cMQAvD_BwE#event-115 (accessed on 19 September 2020). Worldometers.info (accessed on 14 December 2020).

## References

[1] Li Q., Guan X., Wu P., Wang X., Zhou L., Tong Y., Ren R., Leung K.S.M., Lau E.H.Y., Wong J.Y. (2020). Early Transmission Dynamics in Wuhan, China, of Novel Coronavirus–Infected Pneumonia. N. Engl. J. Med..

[2] Zhu N., Zhang D., Wang W., Li X., Yang B., Song J., Zhao X., Huang B., Shi W., Lu R. (2020). A Novel Coronavirus from Patients with Pneumonia in China, 2019. N. Engl. J. Med..

[3] World Health Organization Timeline of WHO’s Response to COVID-19. https://www.who.int/emergencies/diseases/novel-coronavirus-2019/interactive-timeline?gclid=CjwKCAiAl4WABhAJEiwATUnEF5RRrhA4IGUZEpKYWv7jCsg3jdx1A_i1IROWObN_C3YIL7JkYNP5yRoC8cMQAvD_BwE#event-115.

[4] Nussbaumer-Streit B., Mayr V., Dobrescu A.I., Chapman A., Persad E., Klerings I., Wagner G., Siebert U., Ledinger D., Zachariah C. (2020). Quarantine alone or in combination with other public health measures to control COVID-19: A rapid review. Cochrane Database Syst. Rev..

[5] Qian M., Jiang J. (2020). COVID-19 and social distancing. J. Public Health.

[6] World Health Organization (2020). WHO Coronavirus Disease (COVID-19) Dashboard. https://covid19.who.int/.

[7] (2020). COVID-19 Coronavirus Pandemic. Worldometers.info.

[8] Arabia S.N. The Expansion of the Grand Mosque. Milestones and Stages (Arabic).

[9] General Presidency for the Affairs of the Grand Mosque and the Prophet’s Mosque The Architecture of the Prophet Mosque (Arabic). http://wmn.gov.sa/news/5002/4/عمارة-المسجد-النبوي.

[10] Statistics, G.A. for Umrah 2019. https://www.stats.gov.sa/sites/default/files/umrah_2019_a-15-3.pdf.

[11] Hajj Statistics 2019–1440. https://www.stats.gov.sa/sites/default/files/haj_40_en.pdf.

[12] Ministry of Islamic and Mosque Affairs (2019). The Statistical Book of the Financial Year 1438/1439H.

[13] Salimi R., Gomar R., Heshmati B. (2020). The COVID-19 outbreak in Iran. J. Glob. Health.

[14] Tuite A.R., Bogoch I.I., Sherbo R., Watts A., Fisman D., Khan K. (2020). Estimation of Coronavirus Disease 2019 (COVID-19) Burden and Potential for International Dissemination of Infection From Iran. Ann. Intern. Med..

[15] Habibi R., Burci G.L., De Campos T.C., Chirwa D., Cinà M., Dagron S., Eccleston-Turner M., Forman L., O Gostin L., Meier B.M. (2020). Do not violate the International Health Regulations during the COVID-19 outbreak. Lancet.

[16] Kucharski A.J., Russell T.W., Diamond C., Liu Y., Edmunds J., Funk S., Eggo R.M., Sun F., Jit M., Munday J.D. (2020). Early dynamics of transmission and control of COVID-19: A mathematical modelling study. Lancet Infect. Dis..

[17] Chinazzi M., Davis J.T., Ajelli M., Gioannini C., Litvinova M., Merler S., Piontti A.P.Y., Mu K., Rossi L., Sun K. (2020). The effect of travel restrictions on the spread of the 2019 novel coronavirus (COVID-19) outbreak. Science.

[18] Anzai A., Kobayashi T., Linton N.M., Kinoshita R., Hayashi K., Suzuki A., Yang Y., Jung S.-M., Miyama T., Akhmetzhanov A.R. (2020). Assessing the Impact of Reduced Travel on Exportation Dynamics of Novel Coronavirus Infection (COVID-19). J. Clin. Med..

[19] Shereen M.A., Khan S., Kazmi A., Bashir N., Siddique R. (2020). COVID-19 infection: Origin, transmission, and characteristics of human coronaviruses. J. Adv. Res..

[20] Anderson E.L., Turnham P., Griffin J.R., Clarke C.C. (2020). Consideration of the Aerosol Transmission for COVID-19 and Public Health. Risk Anal..

[21] Koo J.R., Cook A.R., Park M., Sun Y., Sun H., Lim J.T., Tam C., Dickens B.L. (2020). Interventions to mitigate early spread of SARS-CoV-2 in Singapore: A modelling study. Lancet Infect. Dis..

[22] Wang X., Pasco R.F., Du Z., Petty M., Fox S.J., Galvani A.P., Pignone M., Johnston S.C., Meyers L.A. (2020). Impact of Social Distancing Measures on Coronavirus Disease Healthcare Demand, Central Texas, USA. Emerg. Infect. Dis..

[23] Kang Y.-J. (2020). Lessons Learned From Cases of COVID-19 Infection in South Korea. Disaster Med. Public Heal. Prep..

[24] Ebrahim S.H., Memish Z.A. (2020). Saudi Arabia’s drastic measures to curb the COVID-19 outbreak: Temporary suspension of the Umrah pilgrimage. J. Travel Med..

[25] Al Mahanadi B.A.H. (2019). The Role of Transport and Tourism Economics in Achieving the Economic Development of the Kingdom of Saudi Arabia for the Period (2007–2017). J. Econ. Adm. Leg. Sci..

[26] World Health Organization (2020). Coronavirus Disease 2019 (COVID-19) Situation Report—72. https://apps.who.int/iris/bitstream/handle/10665/331685/nCoVsitrep01Apr2020-eng.pdf.

[27] Studdert D.M., Hall M.A. (2020). Disease Control, Civil Liberties, and Mass Testing—Calibrating Restrictions during the Covid-19 Pandemic. N. Engl. J. Med..

[28] COVID-19 Outbreak Live Update. https://www.worldometers.info/coronavirus/.

[29] John T.J., Samuel R. (2000). Herd immunity and herd effect: New insights and definitions. Eur. J. Epidemiology.

[30] Cao D., Yin H., Chen J., Tang F., Peng M., Li R., Xie H., Wei X., Zhao Y., Sun G. (2020). Clinical analysis of ten pregnant women with COVID-19 in Wuhan, China: A retrospective study. Int. J. Infect. Dis..

[31] Kamerlin C.L., Kasson P.M. (2020). Managing Coronavirus Disease 2019 Spread With Voluntary Public Health Measures: Sweden as a Case Study for Pandemic Control. Clin. Infect. Dis..

[32] Cabinet Office Face Coverings: When to Wear One and How to Make your Own. https://www.gov.uk/government/publications/face-coverings-when-to-wear-one-and-how-to-make-your-own/face-coverings-when-to-wear-one-and-how-to-make-your-own#face-coverings-at-work.

[33] Xiao Y., Torok M.E. (2020). Taking the right measures to control COVID-19. Lancet Infect. Dis..

